# Evaluation of Scales of Tilapia Sp. and *Sciaenops ocellatus* as Low Cost and Green Adsorbent for fluoride Removal From Water

**DOI:** 10.3389/fchem.2022.813533

**Published:** 2022-03-23

**Authors:** Marian Asantewah Nkansah, Asare Boateng Dua, Gabriel Adjei Aryee, Junias Adusei-Gyamfi

**Affiliations:** ^1^ Department of Chemistry, Kwame Nkrumah University of Science and Technology, Kumasi, Ghana; ^2^ Department of Environmental Science, Kwame Nkrumah University of Science and Technology, Kumasi, Ghana

**Keywords:** adsorption, green adsorbent, fish scales, drinking water treatment, isotherms, defluoridation

## Abstract

Water containing more than 1.5 mg/L of fluoride is considered toxic as it causes dental, kidney, and other health problems. With the purpose of helping alleviate these problems by exploring a treatment method for fluoride contamination, this study was to assess the suitability of scales of Tilapia Sp. and *Sciaenops ocellatus* as a cheaper source of adsorbent for the removal of fluoride from drinking water. The samples which were obtained from the Lapaz Market in Accra, Ghana, underwent treatment to eliminate any impurities. They were then ground into powder and treated with aluminum hydroxide [Al(OH)_3_]. The treated samples were used for the removal of fluoride from spiked solutions prepared in the laboratory. Batch adsorption was performed by varying parameters such as adsorbent dose (1–8 g/L), initial concentration (2 mg/L to 10 mg/L), and contact time (30–300 min) at pH of 7. A one-way ANOVA was used to validate the significance of the defluoridation process with respect to the different experimental conditions. The optimum adsorbent dose, initial concentration, and contact time were found to be 4 g/L, 10 mg/L, and 300 min, respectively. The results revealed that the maximum percentage removal of fluoride was 76% by Tilapia Sp. and 70% by *Sciaenops ocellatus* at the optimum conditions. This is an indication that both Tilapia Sp*.* And *Sciaenops ocellatus* scales are suitable adsorbents for the removal of fluoride from water. The fluoride adsorption kinetics followed the pseudo-second-order model, and the adsorption isotherm fitted the Freundlich Isotherm model better than the Langmuir Isotherm model. The adsorption intensity and adsorption capacity for Tilapia Sp*.* were 3.484 L/mg and 0.065 mg/g, and that of *Sciaenops ocellatus* 3.195 L/mg and 0.045 mg/g respectively.

## 1 Introduction

As of 2017, about 785 million of the global population was estimated to lack basic drinking-water sources, while about 144 million people depended on surface water for survival. These surface waters are often susceptible to contamination, causing various waterborne diseases (WHO, 2019). One natural element which is found in water and has both beneficial and detrimental effects on human health is fluoride. At low levels in drinking water, the presence of fluoride reduces the prevalence of dental caries, while the presence of fluoride in drinking water at high concentrations, causes skeletal and dental fluorosis and bone fractures. This is because whereas only about 50–80% of fluoride ingested from food is absorbed, 100% of fluoride ingested from water is absorbed (Yang et al., 2019). As a result, based on World Health Organization (WHO) guidelines, fluoride present in food or water must not exceed 1.5 mg/L (Valdez-Jiménez et al., 2011; Yang et al., 2019; Srivastava and Flora, 2020).

Children are most affected by fluoride toxicity since their skeletal tissue retains up to 50% of ingested fluoride when compared with that of adults, which is approximately 10%. Fluoride is also known to cross the blood-brain-barrier to accumulate in the brain tissue of a developing fetus to cause learning disorders (Qing-Feng et al., 2019). People experiencing these effects are often found in underdeveloped areas of Asia and Africa. Craig et al. (2015) discovered that in the Bongo District of Ghana’s Upper East Region, the main source of water is underground, and the average amount of water consumed is twice that of the other regions in the country, but had up to 4 mg/L of fluoride in their water. Through speciation analysis, it has been established that fluoride complexes in groundwater are dominated by free fluoride ions (Li et al., 2017). Therefore, it is a necessity to develop methods to reduce the fluoride content of water to alleviate the detrimental effects of fluorosis and avert half of the world’s population from living in water-stressed areas by 2025 (WHO, 2019).

Adsorption has been found to be a convenient method for removing contaminants in water with the use of a wide range of materials as adsorbents. Lavecchia et al. (2012) found alumina rich bauxite (81% Al_2_O_3_) to adsorb 38.5% fluoride as compared to hematite (Fe_2_O_3_.X) which adsorbed only 7.3% fluoride. This was improved (Rafique et al., 2013) by immobilizing the adsorbent, pure alumina on sol-gel to attain an adsorption of 95%. Calcium based adsorbents such as calcite have also proven to be good fluoride adsorbents, with fluoride removal reaching up to 80.6%. Aluminum and calcium-based adsorbents are therefore more promising when compared to iron and some other metals (Rafique et al., 2013; Tomar and Kumar, 2013). Natural sources of these adsorbents are usually minerals such as bauxite, limestone etc., and these are mined, thereby increasing costs. Calcium hydroxyapatite (Ca_5_(PO_4_)_3_OH) is a potential calcium-based adsorbent that can be derived from fish waste such as scales. It proved useful in the removal of Pb from water and was cost effective (Omar et al., 2019). The 2016 Food and Agriculture Organisation (FAO) outlook projected an annual 2–3% increase in fish production thus fish scales serving as adsorbents is an innovative and sustainable solution in managing fish waste since scales, in particular, have low biodegradability (FAO, 2016; Harikrishna et al., 2017). To add to their economic, and environmental benefits as adsorbents, the composition of fish scales which is mainly collagen type I and calcium hydroxyapatite is thermally stable, with freshwater fish scales having the upper hand (Pati et al., 2010). The mechanical support, and immobilization of calcium hydroxyapatite by collagen type I is also a key feature of fish scales (Pati et al., 2010; Gil-Duran et al., 2016).

The adsorption capabilities of several species of fishes’ scales in the removal of a variety of pollutants have been reported in literature (Zhu et al., 2013; Uzunoğlu and Özer, 2016; Bhaumik et al., 2017; Nasiebanda et al., 2020) with fishes like L. rohita, tilapia spp. and oreochromis spp. gaining more research interest probably because of their widespread availability and consumption (Ighalo and Eletta, 2020).

The hydroxyapatite in fish scales is a key component for the adsorption process. The hydroxyl groups are the active sites responsible for pollutant removal *via* adsorption. Fluoride removal is therefore enhanced by increasing the amount of surface hydroxyl groups on the hydroxyapatite by co-precipitation with aluminum hydroxide solution (Nie et al., 2012).

In view of the chemical structure, composition and benefits of using fish scales as adsorbents, this experiment focuses on the removal of fluoride from water using fish scales from two of the most abundant marine and freshwater fish species in Ghana namely *Sciaenops oscellatus* (red drum) and Tilapia sp. The study of the adsorption capacity of *Sciaenops oscellatus,* which is less studied and has limited references in literature makes this study novel, since it provides the opportunity to compare its efficiency with the well-studied *Tilapia sp*.

### 1.1 Characteristics of the Fish Scales From Other Studies

**FIGURE 1:**
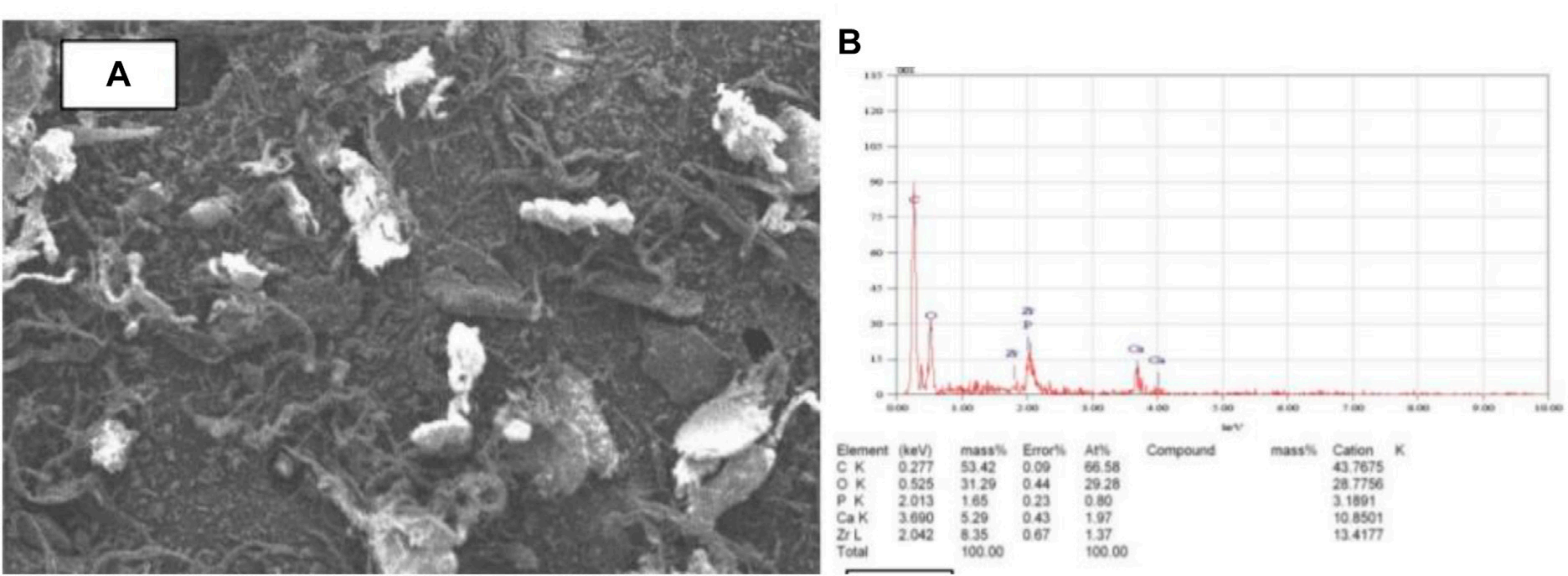
Characterisation of adsorbent **(A)** SEM image for tilapia scales **(B)** EDX analysis of tilapia scales. This figure has been adapted from (Zayadi and Othman, 2013).

The use of scanning electron microscopy (SEM) to observe the surface morphology of the tilapia scales samples by Zayadi and Othman (2013), revealed that the SEM images, had two distinct regions: a bright portion which is rich in inorganic material containing a high proportion of calcium and phosphorus, and a dark region that is rich in protein (Figure 1A). An energy dispersive X-ray analysis (EDX) (Figure 1B), used to identify the elemental composition of the adsorbent showed that carbon is the most dominant element with a mass of about 53% followed by oxygen (31%). Even though the percentage mass of calcium was only about 5%, the X-ray fluorescence (XRF) analysis showed the highest chemical compound was CaO (63.8%) followed by P_2_O_5_ (32.0%). The presence of CaO confirms that high potential of fish scale to adsorb fluoride ions. After defluoridation however, observed SEM images showed structural defects, irregular shape, and coarse surfaces which were attributed to fluoride adsorption by the adsorbents (Nasiebanda et al., 2020). Proximate analysis of scales of red drum fish (*Sciaenops ocellatus*) also revealed that the adsorbent contained about 41% wet wt of crude protein and about 42% wet wt of Ash (Chen et al., 2016).

## 2 Materials and Methods

### 2.1 Materials

Tilapia *Sp*. scales and *Sciaenops ocellatus* scales (from Lapaz market, Accra) were the adsorbents used in this study. Anhydrous sodium fluoride (NaF) served as the source of fluoride, while concentrated hydrochloric acid (HCl) and sodium hydroxide (NaOH) were used for altering pHs. Distilled water was the solvent used in preparing all solutions in this experiment. Aluminium hydroxide was used in the treatment of the scales before use.

### 2.2 Experimental Procedures

Factors such as contact time, adsorbent dosage, adsorbate concentration as well as the Freundlich isotherm and Langmuir adsorption isotherm were used in assessing the rate and mechanism of the adsorption processes. Each experimental condition was repeated thrice.

#### 2.2.1 Treatment of Adsorbent

The adsorbent was prepared by washing with distilled water and drying the sample fish scales. Drying first employed a 3 days solar thermal heating, after which an electric oven (Cole-Parmer instrument company, model: 05015-50) was used at 80°C for 24 h to complete the process. Dried fish scales were then ground with clean porcelain mortar and pestle and sieved to 100 µm with mesh, and was later soaked in 150 ml of 0.1 M of Al (OH)_3_ for 1 h in a shaker (IKA-VIBRAX-VXR) at 220 rpm at room temperature. The adsorbent was then retrieved via filtration and dried in an electric oven at 30 C for 5 h.

#### 2.2.2 Characterization of Fish Scales

FTIR analysis was done to determine the functional groups of the two species of fish scale before and after treatment with aluminium hydroxide solution.

#### 2.2.3 Preparation of Fluoride Solution

A fluoride stock solution of 100 mg/L was prepared from anhydrous NaF for five standard fluoride solutions with concentrations between 2 mg/L and 10 mg/L to be later obtained by dilution using distilled water.

#### 2.2.4 Effect of Adsorbent Dosage

The effect of adsorbent dosage was done using 100 ml of 5 mg/L fluoride solution at pH of 7 in 5,200 ml polyethylene bottles of different batches by varying adsorbent mass. Aliquots of 0.1, 0.2, 0.4, 0.6 and 0.8 g of adsorbent were put into the polyethene bottles, respectively. Bottles were then shaken in a shaker at 220 rpm for 2 hrs and fluoride concentrations were later measured using an ion chromatography (MetrohmHerisau, Switzerland).

#### 2.2.5 Effect of Contact Time Versus Fluoride Concentration

In order to determine the effect of contact time on adsoprtion, residual fluoride concentration was measured at 30, 60, 120, 180, 240, and 300 min respectively. The effect of initial concentration on the adsorption capacity of the adsorbent was studied for optimized conditions of all other parameters by keeping contact time (120 min), adsorbent dose (0.4 g), and pH of 7, and by varying the initial concentration of fluoride solution between 2 and 10 mg/L.

#### 2.2.6 Adsorption Isotherms

Several sorption isotherm models have been extensively used for the modeling of biosorption systems to understand the quantitative relationship between sorbate and sorbent in aqueous phase (Khambhaty et al., 2009).

The Langmuir and Freundlich isotherms were used in this study to explain the adsorption phenomenon. The Langmuir Eq. 1 is commonly written as:

$$\frac{C_{e}}{q_{e}}=\frac{C_{e}}{b}+\;\frac{1}{q_{m}b}$$ (1)

Where, q_e_ is the amount of fluoride adsorbed (mg/g) and C_e_ is equilibrium concentration of adsorbate (mg/L), q_m_ and b are Langmuir constants related to monolayer adsorption capacity and energy of adsorption respectively. The Freundlich Eq. 2 is basically empirical but it is often useful as a means for data description. The general form of Freundlich isotherm is given in the following equation:

$${\text{q}}_{\text{e}}{=\text{K}}_{\text{f}}{\text{Ce}}^{1\text{/n}}$$ (2)

The linearized form of Freundlich isotherm is given by the following Eq. 3.

$$\text{log}\left({\text{q}}_{\text{e}}\right){=\text{log K}}_{\text{f}}{+\text{l/n log C}}_{\text{e}}$$ (3)

The intercept K_f_ is an indicator of sorption capacity, and the slope 1/n is an indicator of sorption strength/intensity and a measure of the deviation from linearity of the adsorption.

#### 2.2.7 Adsorption Kinetics

One of the important factors that controls the rate of adsorbate (fluoride ions) uptake at the solid-liquid interface and the adsorption equilibrium time is the adsorption kinetics (Bhaumik et al., 2017). Different adsorption kinetic models, pseudo-first-order (Eq. 4) and pseudo-second-order (Eq. 5) were applied to the experimental data to obtain the values of the kinetic constants and predicted the equilibrium adsorption capacities.

$$\text{log}\left({\text{q}}_{\text{e}}-{\text{q}}_{\text{t}}\right){=\text{log q}}_{\text{e}}{-\text{K}}_{1}\frac{t}{2.303}$$ (4)

where q_e_ and q_t_ are the amounts of F^−^ adsorbed (mg/g) at equilibrium and at time *t*, respectively, and k_1_ is the adsorption rate constant, which can be determined from the slope of linear plot of log(q_e_ - q_t_) versus *t*.

$$\frac{t}{q_{t}}=\frac{1}{K_{2}q_{e}2}+\;\frac{t}{q_{e}}$$ (5)

$${\text{h}}_{\text{o}}{=\text{k}}_{2}{\text{q}}_{\text{e}}^{2}$$ (6)

where k_2_ is the adsorption rate constant, which can be determined by plotting t/q_t_ versus t, h_o_ represents the initial adsorption rate (Eq. 6), (mg/g/min), and k_2_ is the pseudo-second-order rate constant (g adsorbent/mg adsorbate/min). The slope and intercept of t/(q_t_) vs. t plot gives h_o_ and k_2_ (Adane et al., 2015). The kinetics study at the different contact times was obtained by calculating the instantaneous adsorption capacity, q_e_, following Eq. 7 (Lo et al., 2012):

$${\text{q}}_{\text{e}}=\frac{\left(C_{i}-C_{t}\right)v}{m}$$ (7)

where *C*_*i*_, is the initial concentration of the pollutant, *C*_*t*_ (mg/L) is the residual concentration of pollutant in the liquid phase for each contact time t, *m* is the mass (g) of the adsorbent and *V* representing the volume (L).

## 3 Results and Discussion

**FIGURE 2:**
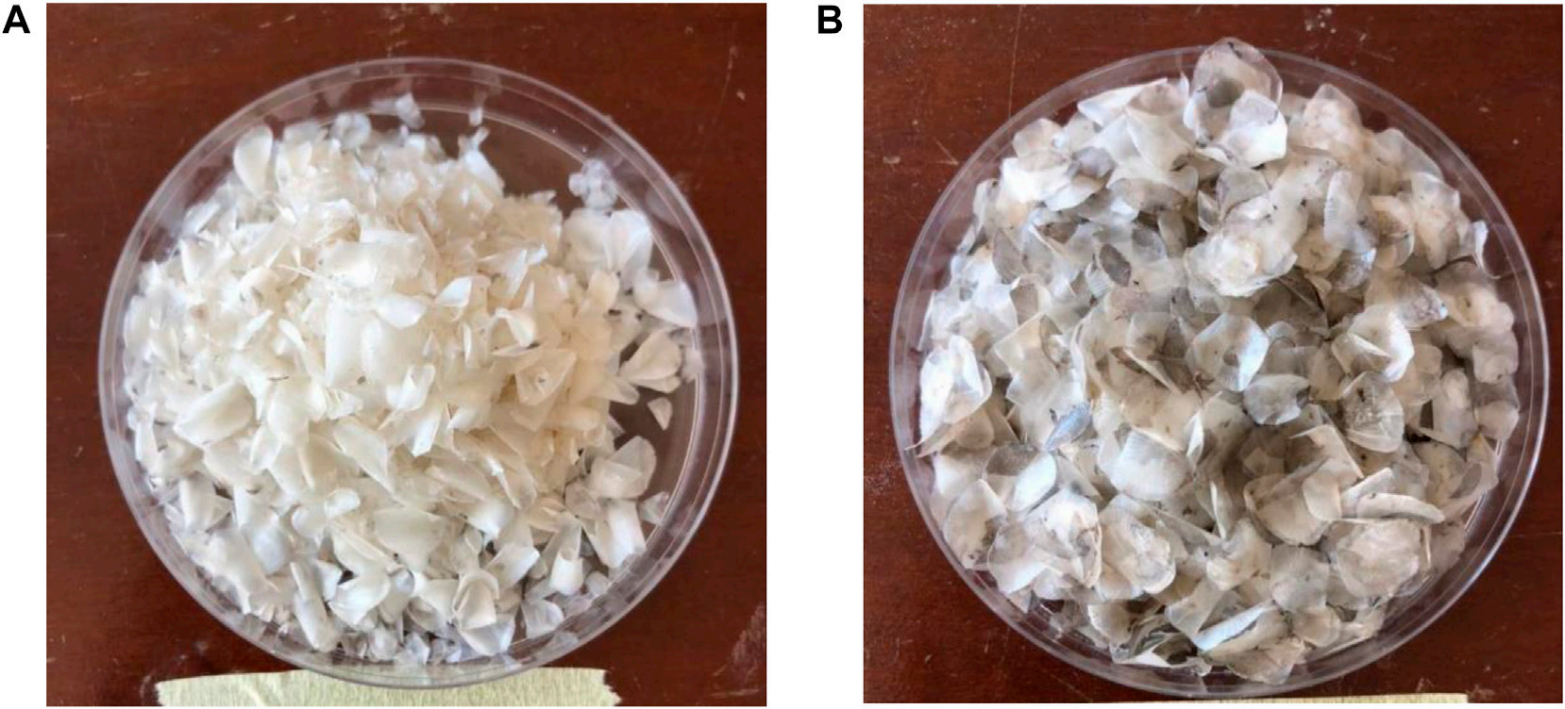
**(A)** Scales of *Sciaenops ocellatus*. **(B)**: Scales of Tilapia Sp.

The scales of *Tilapia Sp*. and *Sciaenops ocellatus* (Figure 2) before and after **Al**(**OH**)_**3**_ treatment have been treated with and characterized for their suitability as adsorbent for fluoride removal and are presented in this section. Data from batch adsorption studies are also illustrated here.

### 3.1 FTIR Spectra of Fish Scales Before and After Treatment With Aluminium Hydroxide

The natural composition of fish scales includes a surface layer which contains hydroxyapatite (Ca_5_(PO_4_)_3_OH), calcium carbonate and a deeper layer which contains collagen type I. minute quantities of elemental Ca, Mg, O, Na and S are sometimes present (Basu and Ajit, 2005; Pati et al., 2010).

**FIGURE 3:**
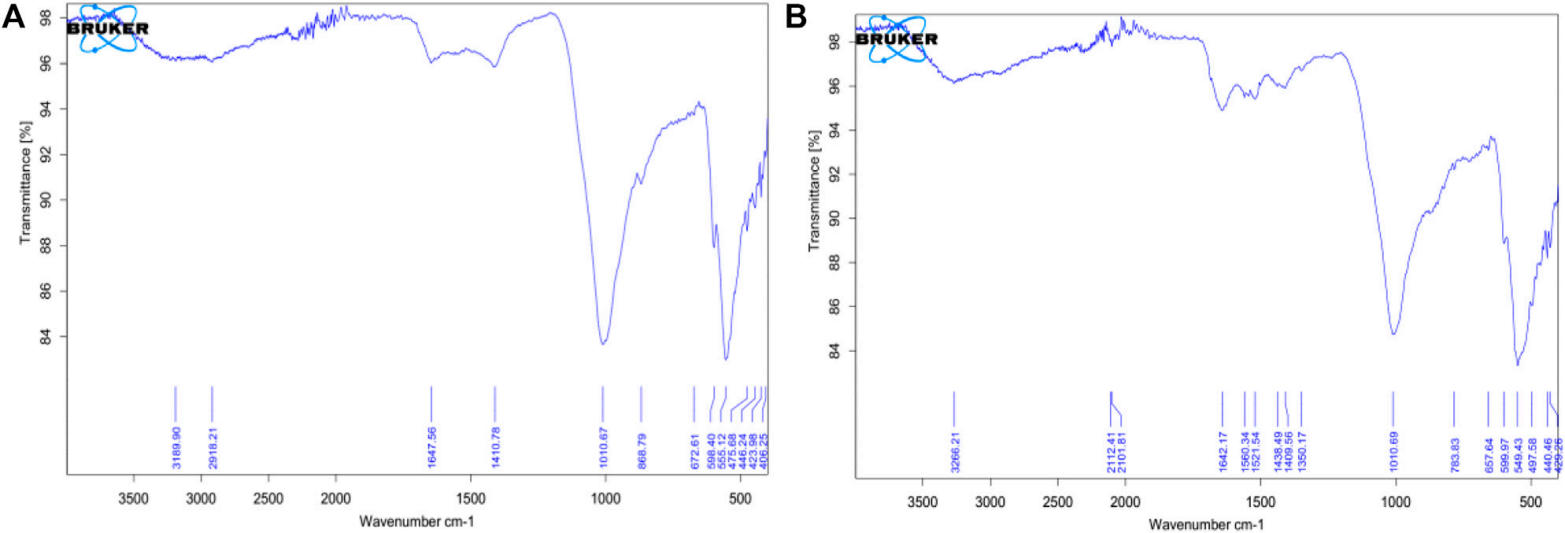
FTIR Spectra of Powdered Scales before chemical treatment (**A**, on the left) Scales of *Sciaenops ocellatus* (**B**, on the right) Scales of *Tilapia* Sp.

From the FTIR data, a characteristic (OH) band around 3,331 cm^−1^ from the hydroxyapatite was expected but this is rather detected at 3,189.90 cm^−1^ and 3,266.21 cm^−1^ for untreated scales of *Sciaenops* and Tilapia Sps. respectively (Figures 3A,B). A similar trend was observed for the spectra of the same scales after treatment with Al(OH)_3,_ with increased wavenumbers of 3,269.20 cm^−1^ and 3,274.32 cm^−1^ respectively (Pati et al., 2010).

**FIGURE 4:**
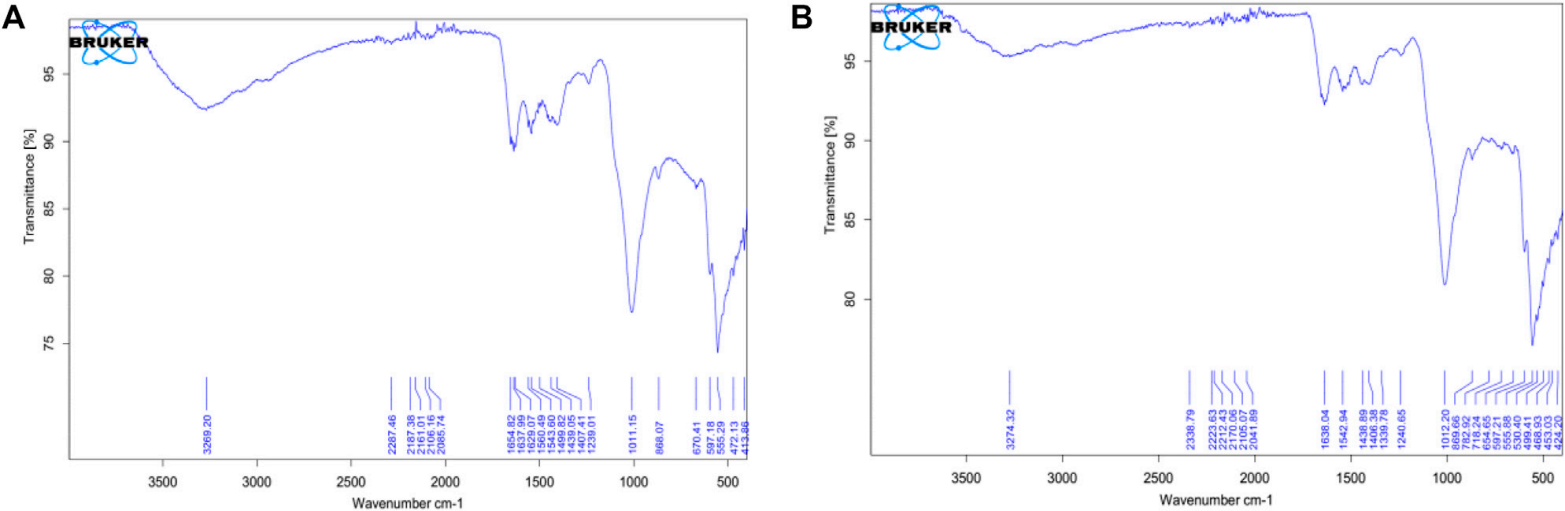
FTIR Spectra of Powdered Scales after chemical treatment (**A**, on the left) Scales of *Sciaenops ocellatus* (**B**, on the right) Scales of *Tilapia* Sp.

Wavenumbers of 1,647.56 cm^−1^ and 1,642.17 cm^−1^ respectively for *Sciaenops* and Tilapia Sps correspond to amide groups of collagen and are associated with stretching vibrations of the –C=O (carbonyl) groups along the polypeptide backbone, which is a sensitive marker of the peptide secondary structure (Figure 3). Similar wavenumbers of 1,654.83 cm^−1^ and 1,638.04 cm^−1^ for *Sciaenops* and Tilapia Sps. Respectively (Figure 4) (Surewicz and Mantsch, 1988).

Fish scale residue has shown a strong absorption peak at about 1,000–1,100 cm^−1^ which was assigned to phosphate group (PO_4_^3−^) stretching, which comes from the C-C stretching of phospholipids in the fish scales and 500–593 cm^−1^ for phosphate group (PO_4_^3−^) bending (Frost et al., 2013; Pourfarzad et al., 2015; Prekajski et al., 2015; Zhang et al., 2019). This trend corresponds to what was observed for the FTIR data for *Sciaenops* and Tilapia Sps. in Figures 3, 4.

The striking differences in the 1,400 to 1,650 cm^−1^ region of the FTIR spectra of *Sciaenops ocellatus* prior to (Figure 3A) and following (Figure 4A) treatment with aluminium hydroxide can be attributed to the removal of non-collagenous proteins from the fish scales. The similarity in the FTIR spectra of both fish scales (Figures 4A,B) after treatment with Al(OH)_3_ indicates untreated *Sciaenops ocellatus* fish scales had more non-collagenous proteins, mainly ichthylepidin (Masood et al., 2014; Sockalingam and Abdullah, 2015).

### 3.2 Effect of Adsorbent Dosage

**FIGURE 5:**
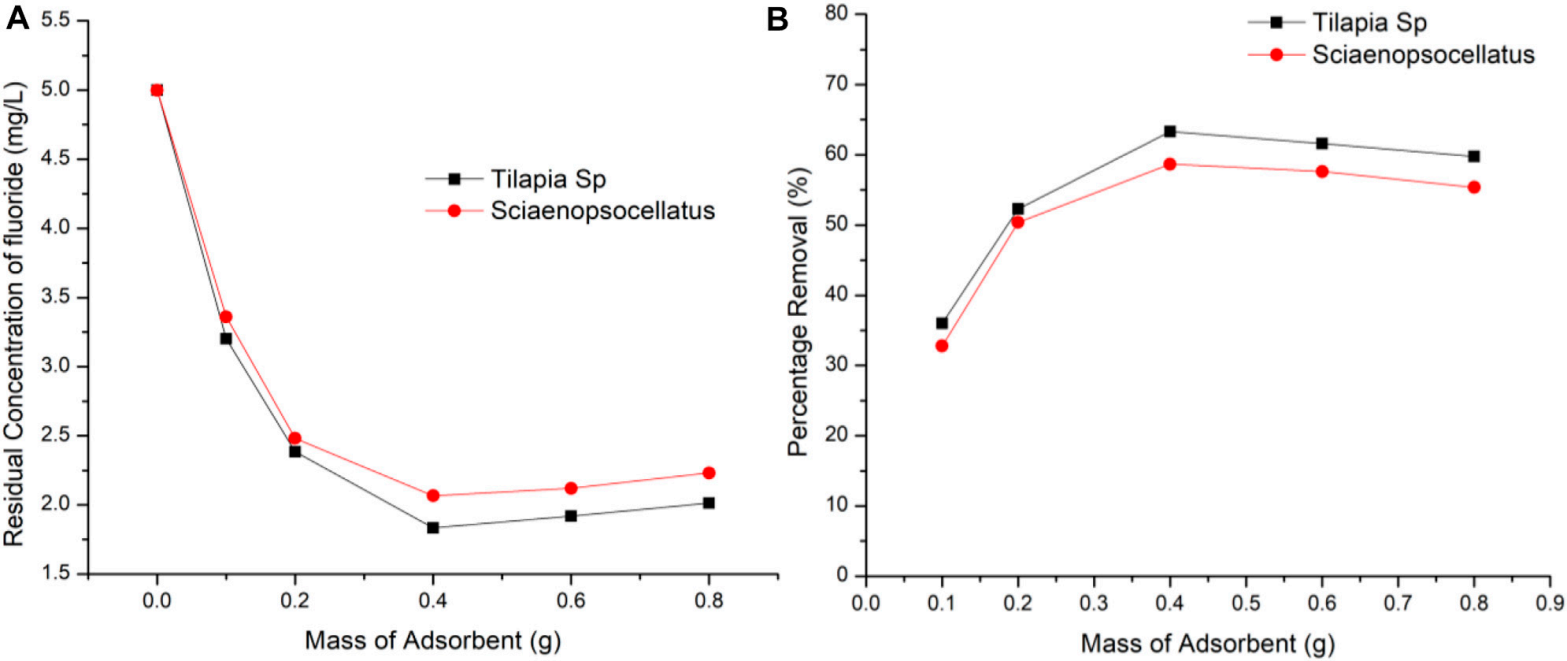
Effect of adsorbent dosage on fluoride removal **(A)** Residual concentration of fluoride **(B)** Percentage removal of fluoride.

At a varying adsorbent dose of 0.1–0.8 g/100 ml and constant fluoride concentration of 5 mg/L the effect of adsorbent dosage was studied using a contact time of 2 h at 220 rpm. The statistical significance of the defluoridation efficiency of the different adsorbent doses was confirmed using a one-way ANOVA, which showed that the computed *p*-value (0.014) was lower than the alpha value (0.05). A possible saturation level of fluoride on Tilapia Sp*.* and *Sciaenops ocellatus* scales was found to occur at 0.4 g dose of adsorbent with scales from Tilapia Sp*.* adsorbing 4.64% more fluoride indicating 0.4 g of Tilapia Sp. scales provides more active sites when compared to *Sciaenops ocellatus scales* (Figure 5)*.* FTIR analysis used to study the adsorption behavior of fluoride have established that fluoride prefers to bind more with the hydroxyl functional group (Mohapatra et al., 2011). Figure 1 shows that there are similar changes in adsorption with respect to adsorbent dose, indicating some similarities between the two adsorbents. As adsorbent dosage exceeds 0.4 g/100 ml it is possible that aggregation occurs thus, inter-particle interaction becomes more prominent, leading to a steady decline in the amount of fluoride removed. The reduction in percentage removal with increasing adsorbent dose which has been reported in previous studies was attributed to the development of aggregates among the adsorbent and the convergence of binding sites, which minimizes the effective active site area (Chowdhury et al., 2011). A similar observation has been reported for the adsorption of fluoride onto Ca-pretreated macrophyte biomass (Miretzky et al., 2008). The possible agglomeration of the adsorbent particles is evidenced by the images of a scanning electron microscope (SEM) which shows morphological defects, irregular shape, and coarse surfaces after fluoride adsorption (Nasiebanda et al., 2020).

### 3.3 Effect of Contact Time

**FIGURE 6:**
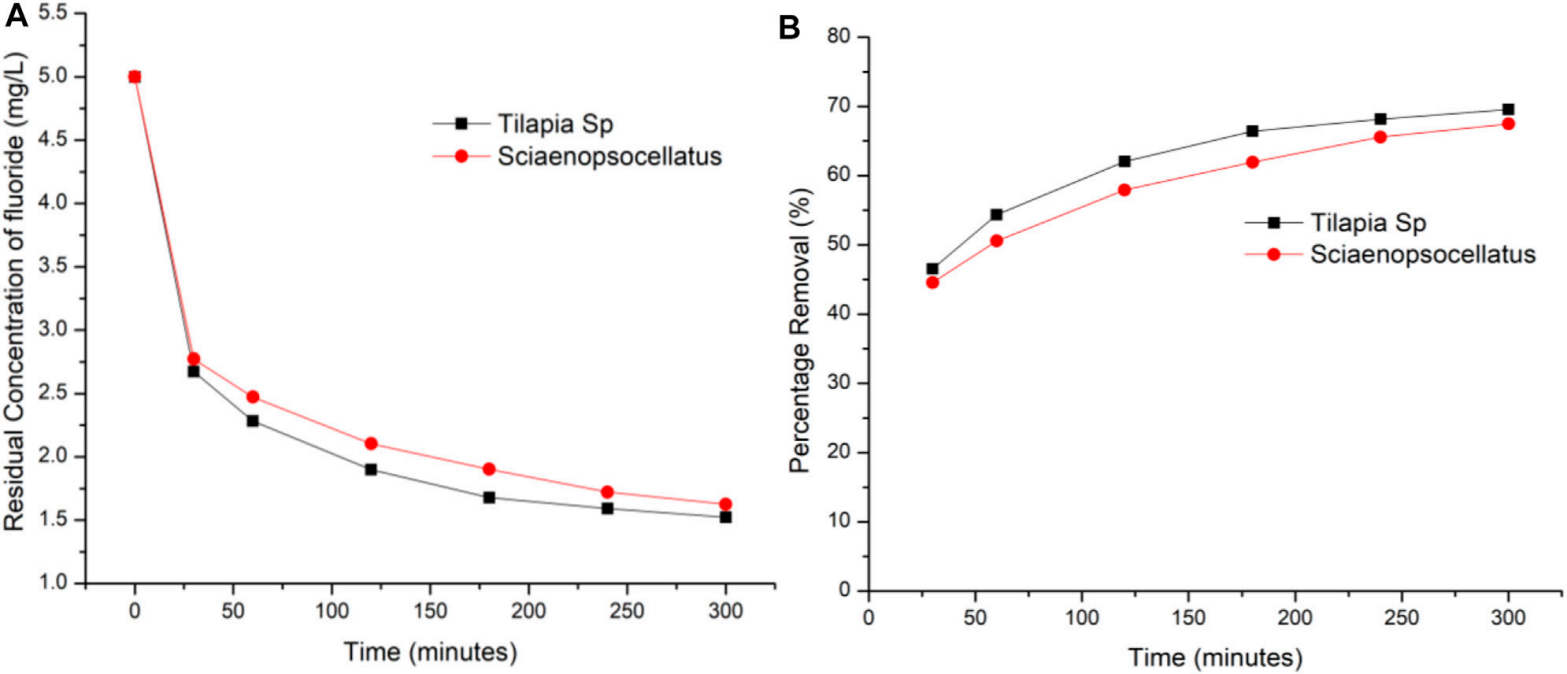
Effect of contact time on fluoride r emoval **(A)** Residual concentration of fluoride **(B)** Percentage removal of fluoride.

Using the optimum adsorbent dosage i.e., 0.4 g/100 ml, the effect of contact time was determined by varying adsorption time from 30 to 300 min with a fixed fluoride concentration of 5 mg/L. Results indicated that there was a constant rise in the amount of fluoride adsorbed on both adsorbents with *Tilapia Sp.* scales always adsorbing 2.48% more fluoride as compared to *Sciaenops ocellatus*. The rate of adsorption of Tilapia Sp*.* within the first 30 min was found to be twice as fast as the proceeding 60 min while that of *Sciaenops ocellatus* was 1.64 times faster (Figure 6). This, when combined with the average percentage difference in adsorption capacity, suggests that Tilapia Sp*.* scales may have more active sites available. The fast adsorption rate at the initial stage has been reported by other studies and attributed to an increased availability in the number of active binding sites on the adsorbent surface (Saha et al., 2010). Figure 2 clearly shows that, the increase in fluoride removal was progressive with time and adsorption equilibrium was seemingly proximate after 300 min since the curve was of logarithmic growth. Adsorption rates and capacities of both adsorbents were also found to remain constant after 300 min of contact time, indicating maximum adsorption capacity was imminent. At 300 min Tilapia Sp*.* scales had a capacity of 69.56% of the initial fluoride while *Sciaenops ocellatus* scales had 67.48%. A similar trend has been observed and reported for the biosorption of fluoride by agronomic product and the defluoridation from synthetic fluoride solution using scales of Indian major carp Catla (Catla catla) (Murugan and Subramanian, 2006; Bhaumik et al., 2017).

### 3.4 Effect of Adsorbate Concentration

**FIGURE 7:**
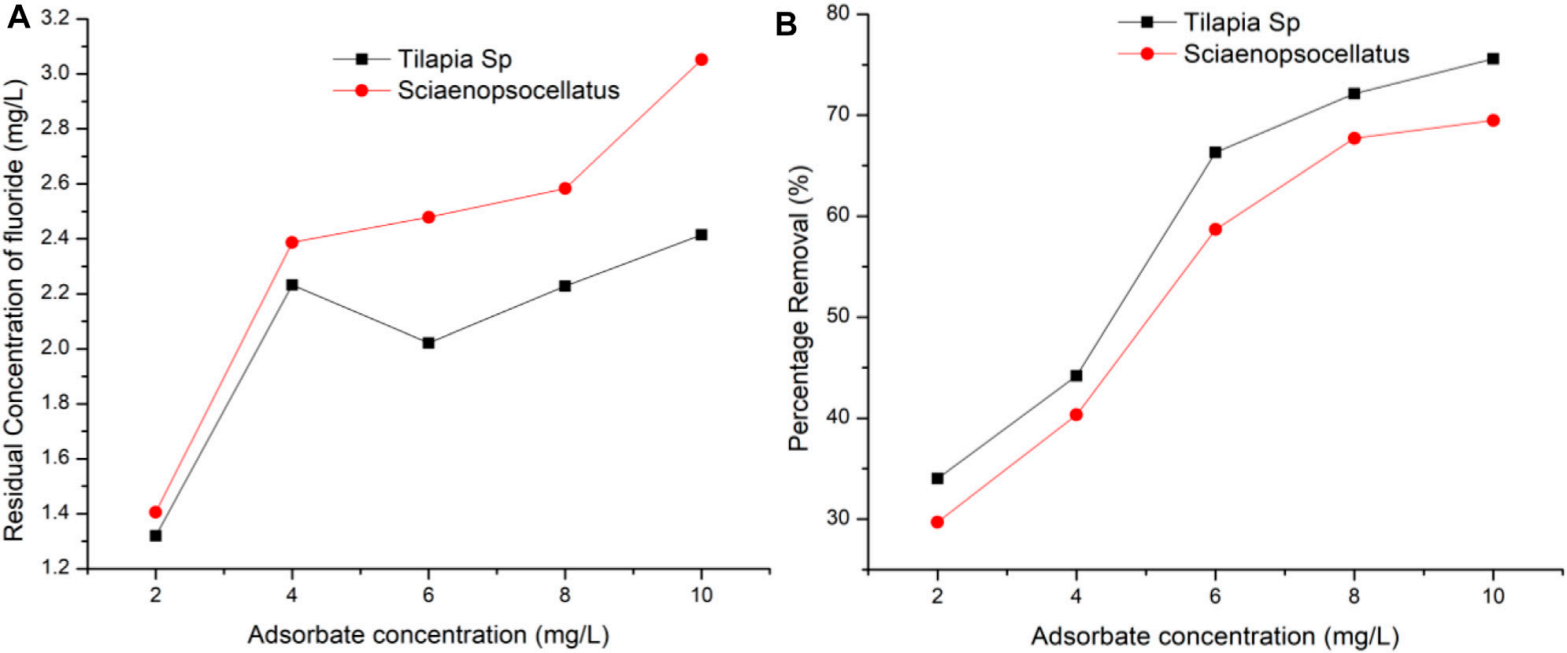
Effect of adsorbate concentration on fluoride removal **(A)** Residual concentration of fluoride **(B)** Percentage removal of fluoride.

At the optimum adsorbent dosage of 0.4 g/100 ml, and fixed contact time of 120 min the effect of increasing adsorbate concentration was found to increase the adsorption on the surface of the adsorbent. From the results, it is clear that the adsorbate was in the least contact with active sites when at 2 and 4 mg/L (Figure 7). Most adsorbates were adsorbed at a concentration of 10 mg/L.

The maximum fluoride removal (89.21%) and uptake capacity (17.84 mg/g) have been reported in other studies to also occur at 10.0 mg/L initial fluoride concentration albeit with different fish species (Catla catla) (Bhaumik et al., 2017). Figure 3, however, indicates more fluoride would have been adsorbed if its concentration was increased beyond 10 mg/L. It is also evident that at all fluoride concentrations, *Tilapia Sp.* scales adsorbed fairly more fluoride as compared to that of *Sciaenops ocellatus.* However, after the adsorption of fluoride has reached in equilibrium, it is expected that the percentage removal of fluoride would decrease with increase in initial fluoride concentration. This is because at higher concentrations, the available attachment sites for a definite amount of adsorbent get saturated (Saha et al., 2010).

### 3.5 Study of Adsorption Isotherms

**FIGURE 8:**
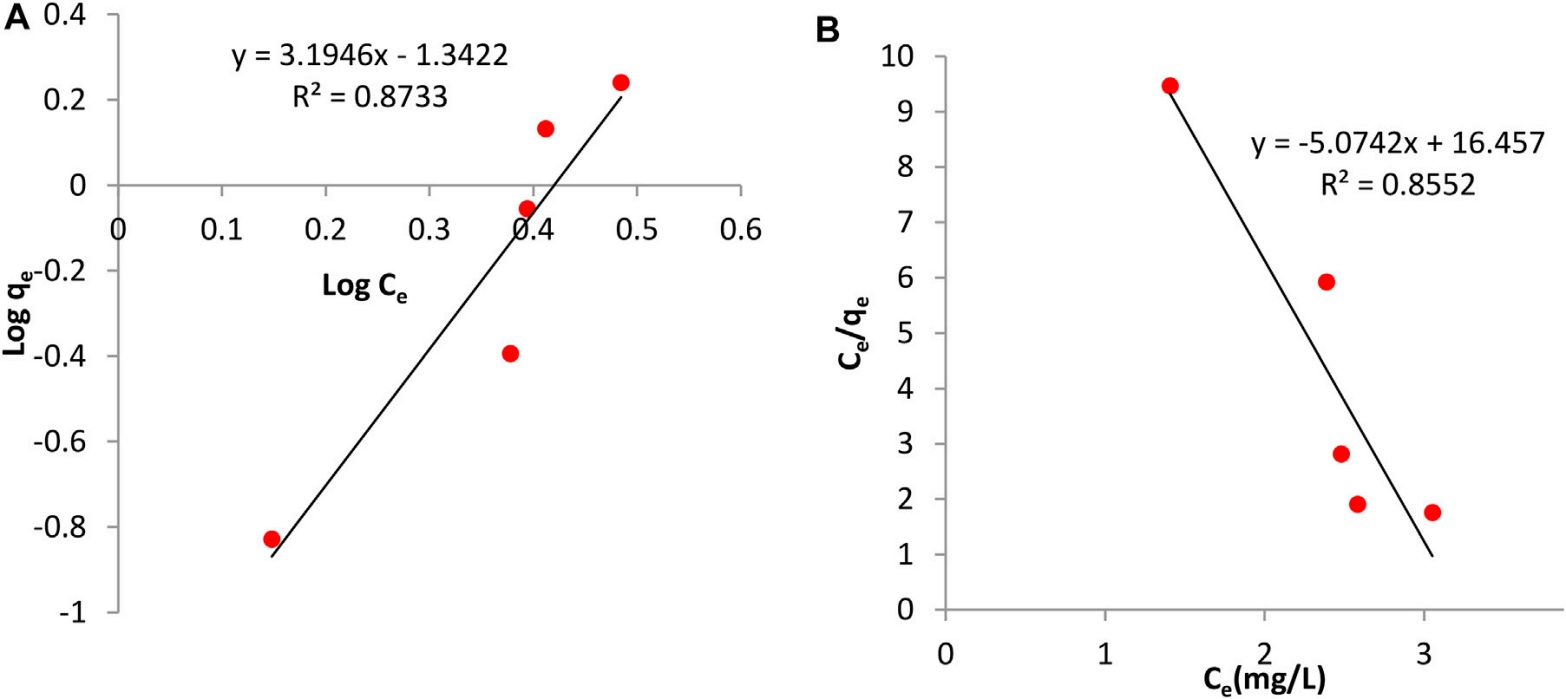
Adsorption isotherms for Sciaenops ocellatus **(A)** Freundlich isotherm **(B)** Langmuir isotherm.

**FIGURE 9:**
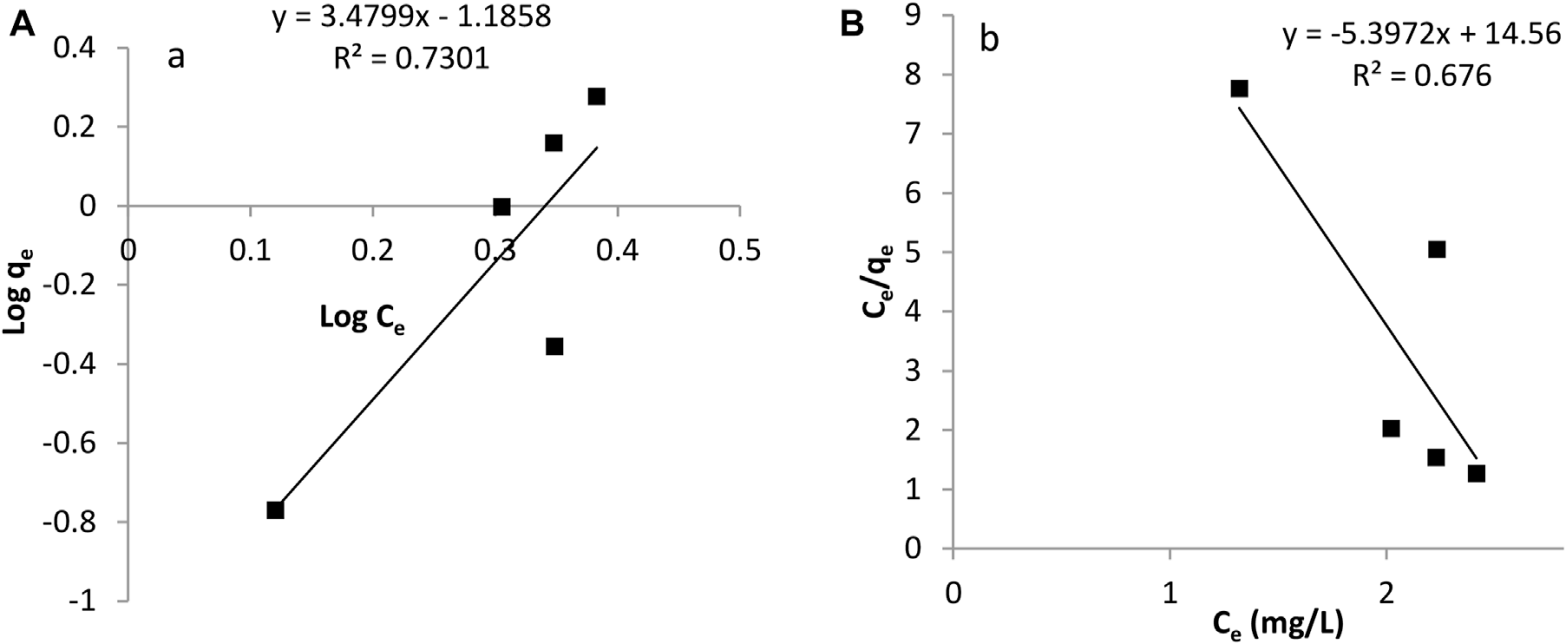
Adsorption isotherms of Tilapia sp. **(A)** Freundlich isotherm **(B)** Langmuir isotherm.

The adsorption isotherm is useful in investigating the feasibility of an adsorbent for an adsorbate. Calculations on the Langmuir and Freundlich isotherm were both conducted. The Langmuir constants, b and monolayer sorption capacity, q_m_ were calculated from the slope and intercept of the plot between C_e_/q_e_ and C_e_ as seen in Figures 8B, 9B. The value of the Langmuir constant, q_m,_ for *Tilapia Sp.* was found to be -0.185 while that of *Sciaenops ocellatus* was -0.197.

The Langmuir constant, b, which denotes adsorption energy for Tilapia *Sp.* and *Sciaenops ocellatus* were found to be -0.371 and -0.308 respectively. Negative q_m_ values indicate the Langmuir isotherm is invalid for studying the adsorption of fluoride on these adsorbents. Therefore, the Freundlich isotherm was employed, and calculations showed the constants 1/n (adsorption intensity) and K_f_ (adsorption capacity) for Tilapia Sp*.* were 3.484 L/mg and 0.065 mg/g respectively and that of *Sciaenops ocellatus* was 3.195 L/mg and 0.045 mg/g. All the sorbents had an exponent (n) lower than one, indicating that these materials (Tilapia Sp. and *Sciaenops ocellatus* scales) could be good fluoride adsorbents. One of the assumptions of the Freundlich multilayer sorption isotherm is that, the amount of adsorbate adsorbed increases infinitely with an increase in concentration (Adeniyi and Ighalo, 2019) which confirms why it was observed in this study that increasing the adsorbate concentration increased the adsorption on the surface of the adsorbent. Additionally, the better fitness of the Freundlich model (see Figures 8A, 9A) as compared to the Langmuir model could be attributed to the heterogeneous surface energies and an exponential distribution of active sites, which are characteristics of the adsorbents. Thus, the stronger binding sites are occupied first, which causes the binding strength to decrease with an increasing degree of site occupation (Adeniyi and Ighalo, 2019).

### 3.6 Adsorption Kinetics

**FIGURE 10:**
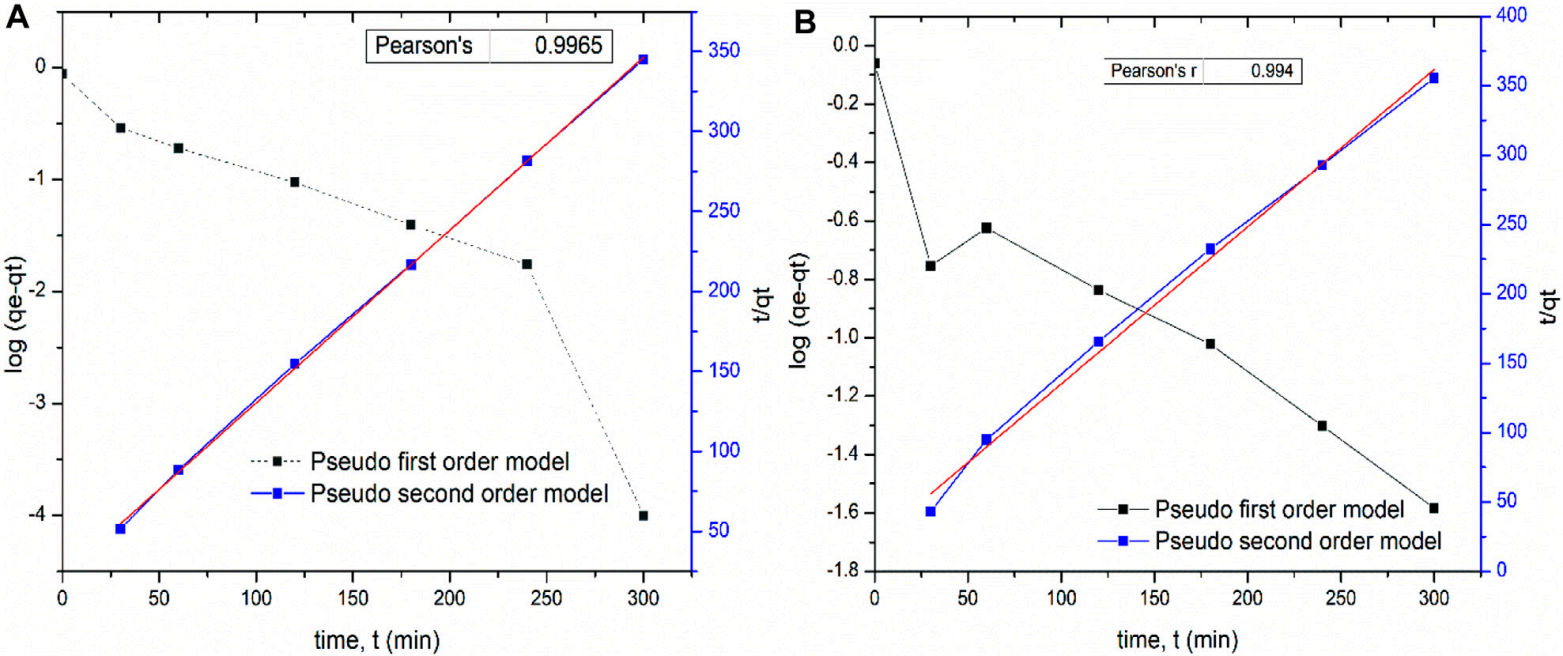
Pseudo-first-order and Pseudo-second-order kinetic plots for the removal of fluoride from solution using scales of (**A**, on the left) Tilapia Sp*.* (**B**, on the right) *Sciaenops ocellatus*.

Comparing the plot and Pearson’s correlation for both models, it was found that the pseudo-second order model for both adsorbents was more linear with a higher correlation coefficients *R* ^2^ = 0.9965 and 0.994 (Figures 10A,B), indicating that this order is more applicable to this study. While the pseudo-second order rate constant for Tilapia sp was 1.119 gmol^−1^ s^−1^, that of Sciaenops ocellatus was 1.172 gmol^−1^ s^−1^. A similar trend of fluoride adsorption on various adsorbents has been reported in other studies (Singh and Cb, 2016; Nasiebanda et al., 2020). If the kinetic model best fits pseudo-first order, it indicates that the reaction is more inclined towards physisorption, and the sorption process only depends on the number of fluoride ions present at a specific time in the solution, whereas if a reaction fits well to the pseudo-second order model, it is assumed that the reaction is more inclined towards chemisorption and the fluoride adsorption process depends on both the number of fluoride ions present in the solution and the free adsorption sites on the biosorbent surface (Bhaumik et al., 2017; Ferreiro et al., 2019). Thus, in this study, it can be concluded that the rate of fluoride adsorption onto the adsorbent was more influenced by surface reactions between the adsorbent sites and fluoride particles than by external transfer processes. Other studies have equally reported fluoride adsorption kinetics following the pseudo-second-order model and the adsorption isotherm fitting Freundlich model better (Bhaumik et al., 2017; Rajkumar et al., 2019).

## 4 Conclusion

The adsorbents produced from treatment of scales Tilapia Sp*.* and *Sciaenops ocellatus* with aluminium hydroxide were found to be efficient for the removal of fluoride ions from water. The optimal adsorbent dosage was 0.4 g/100 ml of fluoride solution, and adsorption was found to be progressive with contact time. With 0.4 g/100 ml of fluoride, it was found that Tilapia Sp*.* scales adsorbed 4.64% more than that of *Sciaenops ocellatus* and the maximum fluoride removed was 63.32%. The difference in the removal efficiencies of both adsorbents was found to be statistically significant based on the ANOVA results. The Langmuir isotherm failed to fit the experimental data indicating the nature of the adsorbent might not satisfy the assumptions of Langmuir but fitted well with the Freundlich isotherm, thus, the surface energies of the adsorbent may be heterogeneous whereas the kinetic model best fitted the second order model. The efficiency of fish scales as fluoride adsorbents was much higher than others such as hematite in other studies. Hence considering the abundance of fish scale as waste, it can serve as a sustainable source of fluoride adsorbent. More research is needed to understand the surface morphology of the adsorbents, especially that of *Sciaenops ocellatus.* The spent adsorbent needs to be generated and properly disposed of. Previous studies have shown that the adsorbed fluoride can be regenerated using NaOH solution. Further studies are therefore required to confirm the feasibility of this regeneration process for the adsorbents used for this study.

## Data Availability

The original contributions presented in the study are included in the article/Supplementary Material, further inquiries can be directed to the corresponding author.
